# Telomere Length Measurement Using CRISPR-Cas12a

**DOI:** 10.1093/geroni/igaf122.4018

**Published:** 2025-12-31

**Authors:** Waylon Hastings, Anna Tillinghast

**Affiliations:** Texas A&M University, College Station, Texas, United States; Texas A&M University, College Station, Texas, United States

## Abstract

**Background:**

Mixed results of meta-analyses have led to questions about the validity of telomere length (TL) as a biomarker of aging. Conflicting findings are in part attributable limitations in TL measurement methodology, which for epidemiological studies is often quantitative-PCR (qPCR). Although high throughput and minimal DNA input make qPCR extremely cost-effective, this comes at the cost of precision and accuracy, which is challenged by the need for non-linear transformations to estimate final TL.

**Methods:**

We outline a pipeline to validate a novel methodology to measure TL using CRISPR-Cas12a that includes quality of control of key reagents, optimization of assay conditions, and experimental design to generate comparative data using qPCR.

**Results:**

Preliminary quality control using gel electrophoresis confirmed length of synthesized oligomer standards. Validation assays further supported methodological design, such that measurements of dsDNA standards were proportional to total telomere content (R2>0.999) and not influenced by oligomer length (p > 0.05) when assessed using either Cas12a or qPCR. Signals from a 2-foldly diluted standard series were each significantly distinct when assessed using Cas12a (p < 0.05) but not qPCR, suggesting Cas12a provides enhanced precision. By contrast, standards with the lowest total telomeric content were not distinguishable from negative controls when assessed by Cas12a (p > 0.05), but were well above background when assessed using qPCR, suggesting the need to further refine the Cas12 assay to establish an acceptable lower limit of detection.

**Conclusion:**

Additional methodological validation is ongoing, including generating measurements on a set of 100+ samples with paired data generated using qPCR, hybridization, and sequencing-based approaches.

